# Molecular Diagnostic of Ochratoxin A With Specific Aptamers in Corn and Groundnut via Fabrication of a Microfluidic Device

**DOI:** 10.3389/fnut.2022.851787

**Published:** 2022-03-24

**Authors:** Deepshikha Shahdeo, Azmat Ali Khan, Amer M. Alanazi, Vivek K. Bajpai, Shruti Shukla, Sonu Gandhi

**Affiliations:** ^1^DBT-National Institute of Animal Biotechnology (NIAB), Hyderabad, India; ^2^Pharmaceutical Biotechnology Laboratory, Department of Pharmaceutical Chemistry, College of Pharmacy, King Saud University, Riyadh, Saudi Arabia; ^3^Department of Energy and Materials Engineering, Dongguk University, Seoul, South Korea; ^4^TERI-Deakin Nanobiotechnology Centre, Sustainable Agricultural Division, The Energy and Resources Institute, TERI Gram, Gurugram, India

**Keywords:** gold nanoparticles, colorimetric assay, Ochratoxin A, microfluidic, aptasensor

## Abstract

Ochratoxin A (OTA) is one of the predominant mycotoxins that contaminate a wide range of food commodities. In the present study, a 36-mer aptamer was used as a molecular recognition element coupled with gold nanoparticles (AuNPs) for colorimetric detection of OTA in a microfluidic paper-based analytical device (μPADs). The μPADs consisted of three zones: control, detection, and sample, interconnected by channels. UV-vis spectroscopy (UV-vis), Dynamic Light Scattering (DLS), and Transmission Electron Microscopy (TEM) were used for characterization of AuNPs and AuNPs/Aptamer. According to the colorimetric assay, limit of detection (LOD) was found to be 242, 545.45, and 95.69 ng/mL in water, corn, and groundnut, respectively. The HPLC detection method achieved acceptable coefficient in standard curves (*r*^2^ = 0.9995), improved detection range, and recovery rates in spiked corn and groundnut samples as 43.61 ± 2.18% to 87.10 ± 1.82% and 42.01 ± 1.31% to 86.03 ± 2.64% after multiple sample extractions and cleanup steps. However, the developed μPADs analytical device had the potent ability to rapidly detect OTA without any extraction pre-requirement, derivatization, and cleanup steps, thus illustrating its feasibility in the animal health sector, agricultural, and food industries.

## Introduction

Mycotoxins are secondary metabolites produced by filamentous fungi, such as Aspergillus, Penicillium, Fusarium, etc., that are toxic to animals and humans upon consumption. Aflatoxin, ochratoxins, fumonisins, patulin, zearalenone, and trichothecenes, including deoxynivalenol and T-2 toxin, are the most prevalent mycotoxins identified in feed (1). Ochratoxin (A, B, and C) are common contaminants of agricultural products, such as barley, beans, coffee, groundnut, corn, oats, rice, wheat, and also percolate into animal products, such as eggs, milk, and meat, including human milk (1, 2). OTA is nephrotoxic, genotoxic, neurotoxic, immunotoxic, and embryotoxic with teratogenic and carcinogenic effects (3). According to the International Agency for Research on Cancer (IARC), OTA has been classified as a group 2B carcinogen (4, 5). It was observed that there was an occurrence of renal tumors when the dietary intake was more than 70 μg/kg of OTA per day in humans (6). In chicken farms, feeding OTA-contaminated feed at a rate of 0.025 g/kg over an extended period of time may result in tumour progression in the liver, kidneys, ureters, or spleen (7).

Several researchers have attempted to develop strategies for rapid and sensitive detection of food-based toxic elements, including mycotoxins using different matrices, nanomaterials, and fluorescence quenchers, including quantum dots, Cu^2+^, Co^2+^, gold nanomaterials, and silica nanoparticles (8–12). However, only a few reports on sensing devices with enhanced performance on real food samples have been published, which may lead to the development of portable commercially viable sensing kits for simple conceptual framework and their application in the diagnosis of food toxicants in corn and groundnut samples. Luan et al. created a colorimetric sensing approach based on aptamers that is label-free and can detect OTA (13). Majdinasab et al. demonstrated the different colorimetric-based techniques, such as Enzyme linked immunoassay (ELISA), Lateral flow assay (LFA), microfluidic devices, and homogenous in-solution strategies (14). Furthermore, to the best of our knowledge, there are relatively few studies published in which the proposed sensing technique was compared with conventional techniques in real food samples (corn and groundnut).

The conventional methods based on Thin Layer Chromatography (TLC), Gas Chromatography (GC), Mass Spectrometry (MS), High-Performance Liquid Chromatography (HPLC) require extensive sample preparation, highly skilled personnel, and are time-consuming (15, 16), which demands the development of a rapid and sensitive method for detection of ochratoxin (OTA). Biosensors are analytical tools that can be used for on-site detection of narcotic drugs, pesticides, antibiotics, bacterial and viral infections, cancer biomarkers, and can also be used to detect pregnancy in the early stage (17–20). Recently aptamers are gaining lot of attention as a biorecognition element for detection of analytes. Aptamers are the single stranded nucleotide in range of 20–200 base pair and has remarkable ability to bind with target molecule such as antibodies, proteins, nucleotide and amino acid (21). Aptamer-based assays are emerging as a promising alternative tool for quick detection of mycotoxin based on an AuNPs/salt aggregation assay (16). Furthermore, AuNPs/aptamer-based assay can be utilize in microfluidic devices and gain ample significance due to their cost effectiveness in recent years.

The microfluidic device was fabricated in the laboratory and combined with an aptamer switching technique to detect OTA in corn and groundnut samples. To the best of our knowledge, for the first time, we have demonstrated a colorimetric sensing method based on the AuNPs/salt aggregation and aptamer switching method in collaboration with a microfluidic device for detection of OTA in corn and groundnut. Along with ease of fabrication, this device was also rapid and cost-effective. Here, the aim of the present study was to measure the detectable limit of OTA in corn and groundnut samples. For this, we have fabricated microfluidic device for colorimetric detection with specific aptamers coupled with gold nanoparticles (AuNPs). Aptamers physically bound to the surface of AuNPs, and displacement occurred in presence of the OTA analyte. UV-vis spectroscopy, DLS, and TEM were used to characterize the biophysical properties of the OTA-AuNPs complex. The AuNPs were incubated with a 36-mer aptamer specific for OTA, and detection of OTA analyte in spiked corn and groundnut samples was performed. For the quantitative analysis of the assay, the absorbance ratio of A_630_ and A_520_, which corresponded to the wavelength of aggregated (gray color) and dispersed (red color) AuNPs, was spectrophotometrically measured. The A_630/A520_ ratio indicated the transition of the dispersed state of AuNPs to the aggregated state in the presence or absence of the analyte. The OTA was prepared in the concentration range of 60 to 2 μg/mL with 545.45 and 95.69 ng/mL limit of detection in spiked corn and groundnut samples via AuNPs based rapid aggregation assay. The developed OTA-aptamer-AuNPs based assay was further tested by a microfluidic device validated by HPLC, can be used for rapid and cost-effective detection of ochratoxin without the requirement of highly sophisticated techniques and skilled labor.

## Materials and Methods

### Chemical and Reagents

Gold chloride (HAuCl_4_), Aflatoxin B1, OTA, trysulfonium hexafluorophosphate salt, propylene glycol monomethyl ether acetate, and negative photoresist were purchased from Merck, Delhi, India. Sodium citrate and sodium chloride were purchased from Sisco Research Laboratories Pvt. Ltd, Delhi, India. The 36-mer aptamer sequence of OTA was 5′-GATCGGGTGTGGGTGGCGTAAAGGGAGCATCGGACA-3′, and 21-mer aptamer sequence of aflatoxin B1 5′-GTTGGGCACGTGTTGTCTCTCTGTGTCTCGTGCCCTTCG CTAGGCCCACA-(3′) was purchased from GCC Biotech (India) Pvt. Ltd, Kolkata, India. Nunc Elisa plates were purchased from ThermoFisher Scientific, Delhi, India. Corn and groundnut samples were purchased from a local supermarket in Hyderabad, India. For HPLC analysis, acetonitrile, water, and acetic acid were purchased at a high purity grade from Merck, Delhi, India. All reagents used for the experiments were of analytical grade.

### Sample and Instruments

Corn and groundnut samples were purchased from the local market at Hyderabad, India. All the experiments were performed in accordance with relevant guidelines and regulations. A UV-visible spectrophotometer (model Systonic model S-925 single beam) was used for measuring the absorbance, and the Antonpaar Litesizer™-500 was used to measure the size and surface charge of AuNPs and AuNPs/aptamer complex. A Perkin Elmer EnSpire Multimode plate reader was used to measure the complete absorbance scan of the samples. The morphological analysis of AuNPs was done by TEM (model number JEOL-JEM 2010). HPLC (model number Thermo Scientific, USA) was used for validation studies with a Capcell Pak C18 column (Shiseido, Munich, Germany).

### Synthesis and Characterization of Gold Nanoparticles

Gold Nanoparticles *(*AuNPs) were prepared with a standard citrate reduction method (22). 50 mL of double distilled water was boiled in an Erlenmeyer flask up to boiling point after the addition of 10% HAuCl_4_, followed by 0.5 mL of 1% sodium citrate. The solution was boiled until the color changed from yellow to blue and wine red at the end. After 20 min, the gold nanoparticles (AuNPs) solution was cooled and stored at 4°C until further use. Further, the confirmation of adsorption of OTA aptamers on the surface of AuNPs were done by incubation of 10 mL AuNPs with 200 nM of OTA aptamers at 4 °C. The synthesized AuNPs and OTA aptamer-AuNPs complexes were characterized with UV-vis spectroscopy, dynamic light scattering (DLS) for hydrodynamic diameter, as well as zeta potential, and TEM analysis.

### Optimization of Salt-Induced Aggregation Assay

The optimization of salt-induced aggregation assay was done by using various concentrations of NaCl (10, 15, 20, 25, 30, 35, 40, 45, 50, 55, and 60 mM) with a fixed volume of AuNPs. The optimum concentration of NaCl that led to the aggregation of AuNPs was obtained by taking the ratio of the absorbance at 630/520 nm. Confirmation of the optimum concentration of NaCl required for aggregation assay was further characterized by UV-vis spectroscopy, A_630/520_, DLS for hydrodynamic diameter, and zeta potential.

### Optimization of OTA Aptamer Concentration

The synthesized AuNPs were incubated with different concentrations of OTA aptamer (400, 200, 175, 150, 125, 100, 75, and 50 nM in 1 × PBS, pH 7.4) and allowed to adhere via physical adsorption for 10 min. A fixed concentration of (40 mM) NaCl was added to the aptamer-coated nanoparticles and further incubated for 5 min. The change in the color was recorded from wine red to blue, followed by gray. Similar characterization was done as mentioned in section Optimization of salt-induced aggregation Assay, and a calibration curve was prepared at A_630/520_ to understand the stages of aggregation.

### Colorimetric Assay for OTA Detection

The AuNPs were coated with an optimal concentration of OTA aptamer (175 nM) and were incubated overnight. Various concentrations of OTA (60, 40, 36, 32, 28, 24, 20, 8, 4, and 2 μg/mL) was prepared and mixed with a fixed concentration of OTA aptamer-AuNPs complex and incubated for 6 min for reaction, followed by the addition of 40 mM NaCl. Different concentrations of Afl B1 (60, 40, 36, 32, 28, 24, 20, 8, 4, and 2 μg/mL) were prepared and incubated with the OTA aptamer. The change in the color was recorded from wine red to gray and further characterized by UV-vis spectroscopy, A_630/520_, DLS. The specificity of the OTA aptamer was evaluated with aflatoxin B1 (Afl B1).

### Preparation and Fabrication of Microfluidic Device for Detection of OTA

Graphic design software (Autodesk AutoCAD) was used to create the microfluidic paper device. 5% °*v/v* trysulfonium hexafluorophosphate salt, 43% °*v/v* propylene glycol monomethyl ether acetate and 52% °*w/w* negative photoresist were dissolved and uniformly distributed on the surface of filter paper and kept at room temperature for 5 min. The paper was baked at 60°C for 5 min and allowed to cool before being cleaned with acetone. Furthermore, OTA aptamer-coated AuNPs were immobilized on the detection and control zones. The samples were placed onto the sample zone with and without OTA and allowed to react with the OTA-aptamer-coated AuNPs.

### Real Sample Analysis and HPLC Comparison

To evaluate the feasibility of the proposed assay, corn flour and groundnut were purchased from the market in Hyderabad, India. The groundnuts were crushed to form a complete powder. Corn flour and groundnut powder (10 mg) was mixed with double distilled water (1 mL) separately and spiked with OTA (60, 40, 36, 32, 28, 24, 20, 8, 4, and 2 μg/mL) and allowed to dry for 3 h at 60°C. All the samples were extracted in extraction solvent (methanol:water: 80:20, *v/v*), washed with double distilled water, and filtered through a 0.25 μm filter. One milliliter of filtrate was diluted with 10 mL of water and used directly for the detection. The quantitative analysis of OTA content was carried out using HPLC with a UHPLC system (Thermo Scientific, USA) equipped with a fluorescence detector (standardized at excitation wavelength 330 nm and emission wavelength 460 nm) and a Capcell Pak C18 column (Shiseido). As a mobile phase, a solvent mixture of acetonitrile: water: acetic acid (99:99:2) was pumped at a flow rate of 0.1 mL/min, and the column temperature was maintained at 36 °C. OTA standard solutions of different concentrations (2, 5, 10, 20, 40, and 60 μg/mL) and sample solutions of OTA extract were filtered through a 0.25 μm filter prior to HPLC analysis, and 10 μL of each sample was injected for 20 min (run-time). Analysis of chromatograms was performed by comparisons with the standard curve of OTA.

## Results and Discussion

### Salt Induced Aggregation Assay of OTA

**Figure 1:**
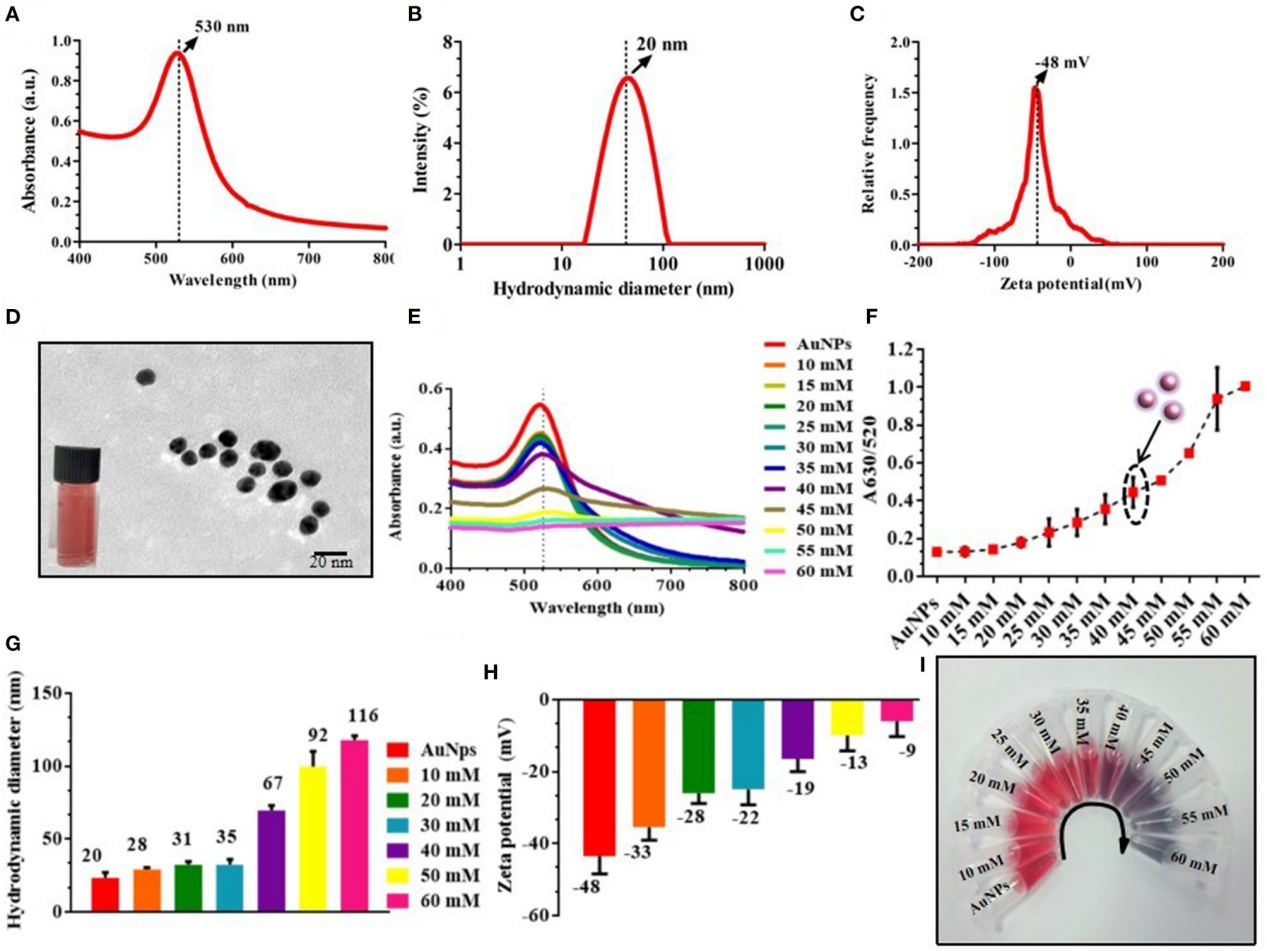
Optimization and characterization of AuNPs and salt-induced aggregation of AuNPs; **(A–C)** UV-vis spectra (530 nm), hydrodynamic diameter (20 nm), and zeta potential (−48 mV) of AuNPs; **(D)** TEM image of monodispersed AuNPs (20 ± 5 nm); **(E)** UV-vis absorbance spectra of AuNPs with different concentration of NaCl; **(F)** A_630/520_ absorbance ratio of different concentrations of NaCl aggregated AuNPs; **(G)** Hydrodynamic diameter of NaCl-induced aggregated particles represents an increase in the size due to aggregation; **(H)** Zeta potential indicates that with an increase in the concentration of NaCl, stability was decreased due to an increase in the surface charge from −48 mV to −9 mV. **(I)** Photographic image of the different concentrations of NaCl with AuNPs.

Detection of OTA was based on the equilibrium between the NaCl-induced aggregated AuNPs and non-aggregated AuNPs. Consequently, to optimize the concentration of NaCl, 20 ± 5 nm size, AuNPs were prepared, which was confirmed with the UV-vis spectra at 530 nm, and hydrodynamic diameter of 20 ± 5 nm (Figures 1A,B) followed by measurement of zeta potential at −48 ± 5 mV and TEM image (Figures 1C,D). Further, a fixed concentration of AuNPs was incubated with different concentrations (10, 15, 20, 25, 30, 35, 40, 45, 50, 55, and 60 mM) of NaCl. The transition of monodispersity to aggregation of AuNPs was also observed as depicted in Figure 1E, where the absorbance intensity of the samples decreased with an increase in the concentration of NaCl along with an increase in the absorbance peak ratio at A_630/520_ (Figure 1F), indicated the aggregation of AuNPs. It was observed that AuNPs were monodispersed up to 35 mM NaCl concentration, while a further increase in the concentration led to aggregation. Furthermore, aggregation was also confirmed with an increase in the hydrodynamic diameter from 20 nm to 116 nm with an increased zeta potential from −48 to −9 mV (Figures 1G,H). Finally, 40 mM of NaCl concentration was considered as an optimum concentration of NaCl for aggregation of the AuNPs (Figure 1I).

### Optimization of OTA Aptamer Concentration With NaCl

**Figure 2:**
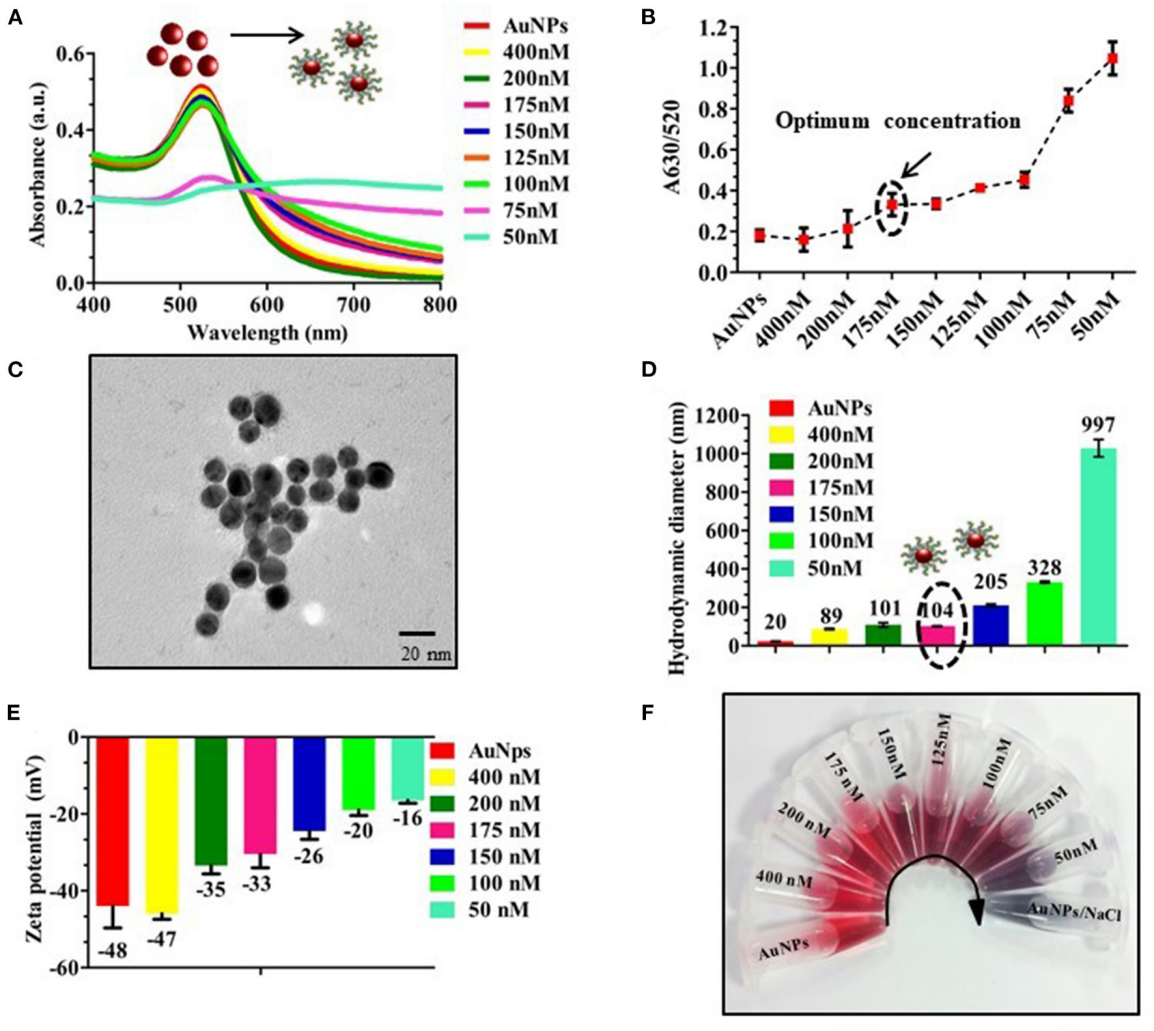
Optimization of aptamer concentration; **(A)** UV-vis spectra of different concentrations of OTA aptamers (400–50 nM) with a fixed volume of AuNPs; **(B)** A_630/520_ absorbance ratio indicating an increase in the aggregation with a decrease in the concentration of aptamer (175 nM) in the presence of 40 mM of NaCl; **(C)** TEM image of OTA aptamer coated AuNPs, a very thin layer of OTA aptamers was observed around AuNPs; **(D,E)** Hydrodynamic diameter and Zeta potential of different concentration of OTA aptamers (400–50 nM) conjugated AuNPs; **(F)** Photographic image of the vials with different concentrations of OTA aptamers.

To optimize the concentration of OTA aptamer to protect AuNPs against NaCl aggregation, AuNPs were initially incubated with different concentrations of aptamer (400–50 nM) to aid proper adsorption of the aptamer onto the surface of AuNPs. From Figure 2A, it is observed that OTA aptamer concentration up to 175 nM resulted in a remarkable resistance against NaCl, and with a further decrease in aptamer concentration, there was an increase in the aggregation of AuNPs along with a shift of 6 nm in UV-vis spectra (from 530 to 536 nm). The absorbance ratio of A_630/520_ (Figure 2B) supported the absorbance spectra as well as naked-eye observation. The coating of OTA aptamer around AuNPs was confirmed with the TEM image (Figure 2C). An increase in the size with the addition of aptamer was also confirmed with an increase in the hydrodynamic diameter of AuNPs from 20 to 104 nm at 175 nM as an optimum concentration of OTA aptamer (Figure 2D). Therefore, the enhanced tolerance toward the NaCl aggregation proved the successful adsorption of OTA aptamers on the surface of AuNPs, which was also clearly indicated by an increase in the zeta potential values from −48 to −16 mV (Figure 2E). A fixed volume of 40 mM NaCl was added to the prepared dilution, and a color change was observed at 150 nM concentration of OTA aptamer (Figure 2F).

### Analytical Detection of OTA

**Figure 3:**
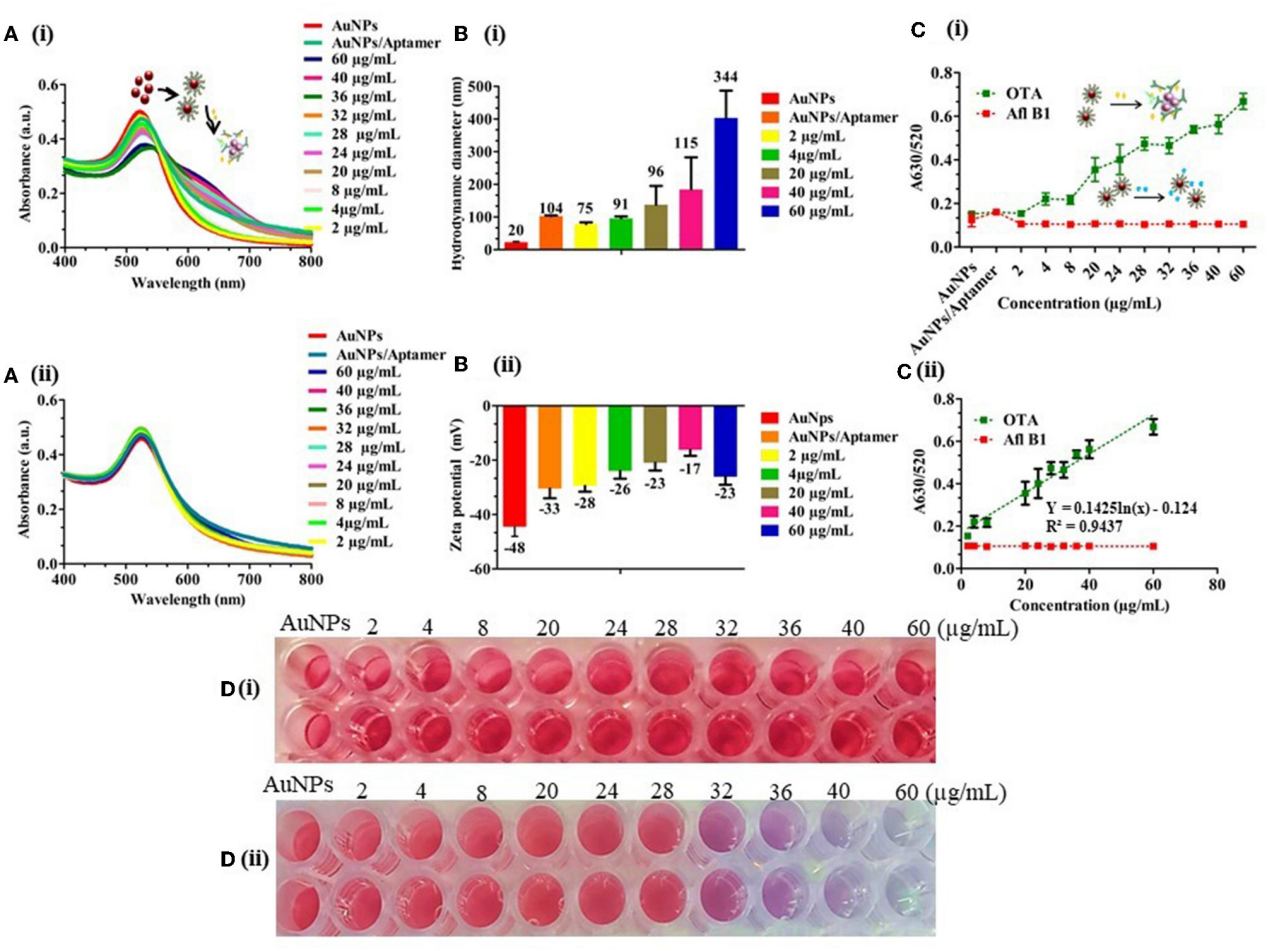
Detection of OTA in spiked water samples; **(A)** (i) and (ii) UV-vis spectra of different concentrations of OTA and Afl B1 (60–2 μg/mL) with OTA aptamer; **(B)** (i) and (ii) Hydrodynamic diameter and Zeta potential of the same which indicated decreased stability with an increased surface charge from −48 to −23 mV; **(C)** (i) A_630/520_ of OTA and Afl B1; **(C)** (ii) Calibration curve of A_630/520_ of OTA and Afl B1; **(D)** (i) and (ii) Photograph of different concentrations of OTA and Afl B1 with OTA aptamer.

Dilutions of OTA (60–2 μg/mL) were prepared and added to different tubes with a fixed aptamer concentration (175 nM) followed by incubation for 5 min at RT. 40 mM NaCl was added to the OTA aptamer adsorbed on the surface of AuNPs and was observed for a change in color. The resulting solutions were transferred into 96-well plates, and absorbance was measured in the range of 400 to 900 nm. A calibration curve was plotted using the A_630/520_ ratio. Absorbance data indicated that with increase in the concentration of analyte (OTA), aggregation was increased, confirming the disassociation of the aptamer from the surface of AuNPs (Figure 3A[i]), while there was no significant shift in the absorbance spectra (Figure 3A[ii]). From the data, we herein calculated the limit of detection (LOD) as 242 ng/mL with visual detection up to 32 μg/mL following the formula: LOD = (3 × standard deviations of blank sample/slope of the calibration curve). An increase in the hydrodynamic diameter of AuNPs from ±20 to ±344 nm (Figure 3B[i]) and zeta potential of aggregated AuNPs from −48 to −23 mV (Figure 3B[ii]) indicated the aggregation of AuNPs with an increased OTA concentration. A_630/520_ also indicated a relative increase in the peak with an increase in the concentration of the target OTA molecule than non-specific Afl B1. The good linear relationship of y = 0.1425ln(x) – 0.124, R^2^ = 0.9437 between the A_630_/A_520_ ratio was obtained (Figure 3C[i,ii]). To evaluate the specificity, OTA aptamers, were incubated with different concentrations of aflatoxin B1 (Afl B1) similarly as used for OTA. It was observed that there was no significant change in the color of Afl B1 as compared to the target OTA (Figure 3D[i,ii]). This demonstrated that the colorimetric assays showed a good specificity.

### Comparison With Conventional HPLC Technique for OTA Detection in Real Samples

**Figure 4:**
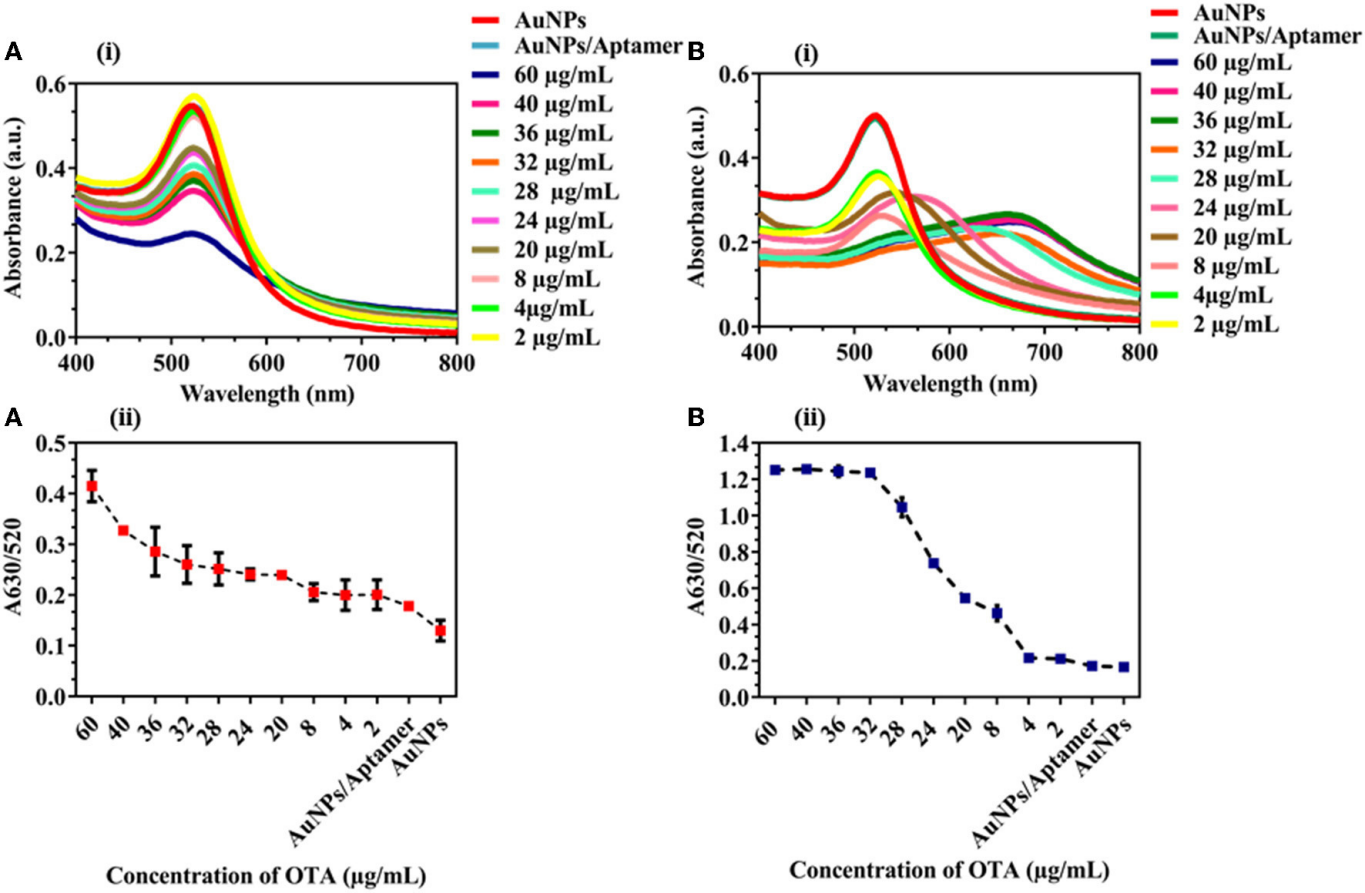
Detection of OTA in corn flour and groundnut: **(A)** (i) and (ii) UV-vis spectra and A_630/520_ nm absorbance peak ratio of corn flour sample spiked with OTA **(B)** (i) and (ii) UV-vis spectra and A_630/520_ nm absorbance peak ratio of groundnut samples spiked with OTA. We observed an increased aggregation tendency with an increase in the concentration of OTA in corn flour and groundnut.

To further evaluate the applicability of the proposed assay, spiked samples of corn flour and groundnut powder were used for real sample analysis. Different concentrations of OTA (60, 40, 36, 32, 28, 24, 20, 8, 4, and 2 μg/mL) were added to the samples with already optimized conditions. A shift in the UV-vis spectra was observed with an increase in the concentration of OTA in spiked samples of corn (Figure 4A[i]) and groundnut (Figure 4A[ii]). A_630/520_ showed an increase in the aggregation with an increase in the concentration of OTA (Figure 4B[i,ii]) in both the samples. The LOD was 545.45 ng/mL and 95.69 ng/mL for corn flour and groundnut samples, respectively. According to the European regulation, Maximum residual limit (MRL) for OTA in cereals to be 5–50 ng/mL [Regulations (EC) No. 1881/2006]. Though the developed microfluidic device is exceeding the MRL value but it has the potential for rapid and on-site detection in comparison with conventional techniques.

**Figure 5:**
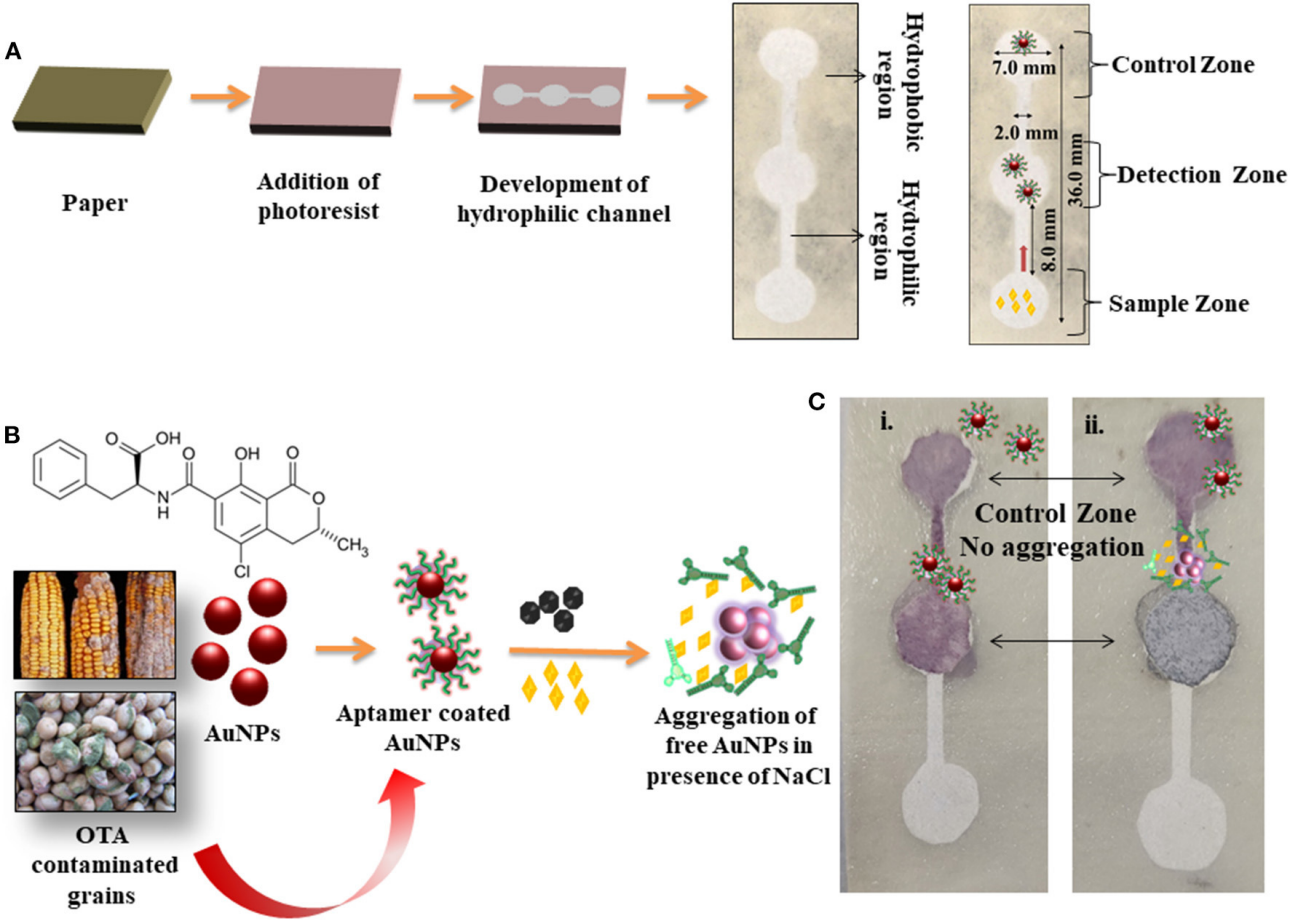
Overview of the development of microfluidic device and detection of OTA; **(A)** Steps depicting the preparation of microfluidic device, generation of hydrophilic channels, and detection zones; **(B)** Chemical formula of OTA and scheme of colorimetric detection of OTA in the grains via aptamer switching technique; **(C)** Colorimetric detection of OTA in a microfluidic device in the **(i)** absence and **(ii)** presence of OTA.

A microfluidic paper device was fabricated using Whatman filter paper. It consisted of a hydrophobic region created with negative photoresist with hydrophilic channels. The device mainly consisted of three zones called detection, sample, and control zone. The hydrophilic zone for sample movement consisted of 7.0 mm diameter, 2.0 mm width, and height 36.0 mm. The detection zone and control zone comprised AuNPs coated with OTA aptamer. The sample zone was loaded with the analyte (OTA) and allowed to move toward the detection zone via a microfluidic channel (Figure 5A). Figure 5B shows the mechanism of colorimetric detection. In the presence of OTA, the AuNPs/OTA aptamer detection zone showed aggregation (color changed to dark gray), while no aggregation was observed in the absence of OTA (Figure 5C). The displacement reaction was considered to be the possible explanation of aggregation due to the specific binding of OTA aptamers with OTA. Therefore, the developed microfluidic device can be further used for the detection of a wide range of toxins and possibly used as an alternative to conventional time consuming and expensive devices. Table 1 shows the comparison of colorimetric and fluorescence-based assays for the detection of mycotoxins in food samples and water. The majority of aptamer-based approaches were developed to detect ochratoxin in red wine, beer, and water by using ELISA, or electrochemical platforms or fluorescence based methods. The developed methods hold promising for high sensitivity but lacking in terms of its field applicability and cost. This is the first time, to the best of our knowledge, that a paper-based microfluidic device (pocket friendly) has been employed for detection of OTA in corn and groundnut as a cost-effective and rapid detection (within 5 min) approach with simple Yes or No concept.

**Table 1:** Comparison between the colorimetric and fluorescence-based assay for the detection of OTA in food samples and water.

**No**.	**Name of toxin**	**Sample**	**Type of technique/ Bio recognition element**	**Limit of detection (LOD)**	**References**
1.	Ochratoxin A	Red wine	AuNPs/Aptamer based sensing	2.08 ng/mL	(23)
2.	Ochratoxin A	Red wine	PVP-coated graphene oxide	8.8 ng/mL	(24)
3.	Ochratoxin A	Red wine	Fluorescent aptasensor/Copper nanoparticles	0.8 ng/mL	(25)
4.	Ochratoxin A	Red wine	Fluorescent quenching/AuNPs	0.07 ng/mL	(26)
5.	Ochratoxin A	Ginger powder	Aptamer/PVP-coated gold nanoparticles	2.01 ng/mL	(27)
6.	Ochratoxin A	Water	Aptamer based bio sensing/AuNPs	8.0 ng/mL	(28)
7.	Ochratoxin A	Beer	Single-walled carbon nanotubes/fluorescent Aptasensor	9.7 ng/mL	(29)
8.	Ochratoxin A	Rice	Gold nanoflowers-based ELISA-colorimetric assay	8.205 pg/mL	(30)
9.	Ochratoxin A	Water	AuNP/Aptamer based colorimetric assay	242 ng/mL	This work
10.	Ochratoxin A	Corn	AuNP/Aptamer based colorimetric assay	545.45 ng/mL	This work
11.	Ochratoxin A	Groundnut	AuNP/Aptamer based colorimetric assay	95.69 ng/mL	This work

To authenticate and validate the feasibility of the developed aptamer and salt-induced AuNP-based colorimetric OTA sensor with respect to its sensitivity, sampling, and sample pre-treatment process, we compared its efficacy with the conventional HPLC approach using the ethyl acetate extraction and cleanup processes (31). Different concentrations of OTA (2, 5, 10, 20, 40, and 60 μg/mL) were used to spike the corn and groundnut samples, followed by extraction and cleanup processes prior to HPLC. OTA recovery levels were monitored based on HPLC chromatograms and calculated via a standard curve equation that was found to fall within the range of 43.61 ± 2.18% to 87.10 ± 1.82% (for corn) and 42.01 ± 1.31% to 86.03 ± 2.64% (for groundnut) (Table 2).

**Table 2:** Recovery of OTA from spiked corn flour and groundnut samples using HPLC.

**Real sample matrices**	**Spiked OTA concentration (μg/mL)**	**Recovered concentration**	**Average recovery**	**% CV**
Corn flour	2	ND	ND	ND
	5	2.18 ± 1.16	43.61 ± 2.18	0.87
	10	6.51 ± 2.31	65.11 ± 0.76	0.94
	20	14.78 ± 1.22	73.90 ± 3.27	1.56
	40	34.52 ± 1.18	86.32 ± 2.26	1.83
	60	52.20 ± 3.30	87.10 ± 1.82	2.22
Groundnut	2	ND	ND	ND
	5	2.10 ± 1.43	42.01 ± 1.31	1.821
	10	5.84 ± 1.11	58.40 ± 1.43	1.65
	20	13.28 ± 4.32	66.40 ± 1.22	2.87
	40	34.16 ± 3.44	85.40 ± 2.19	3.21
	60	51.62 ± 3.13	86.03 ± 2.64	3.69

**Figure 6:**
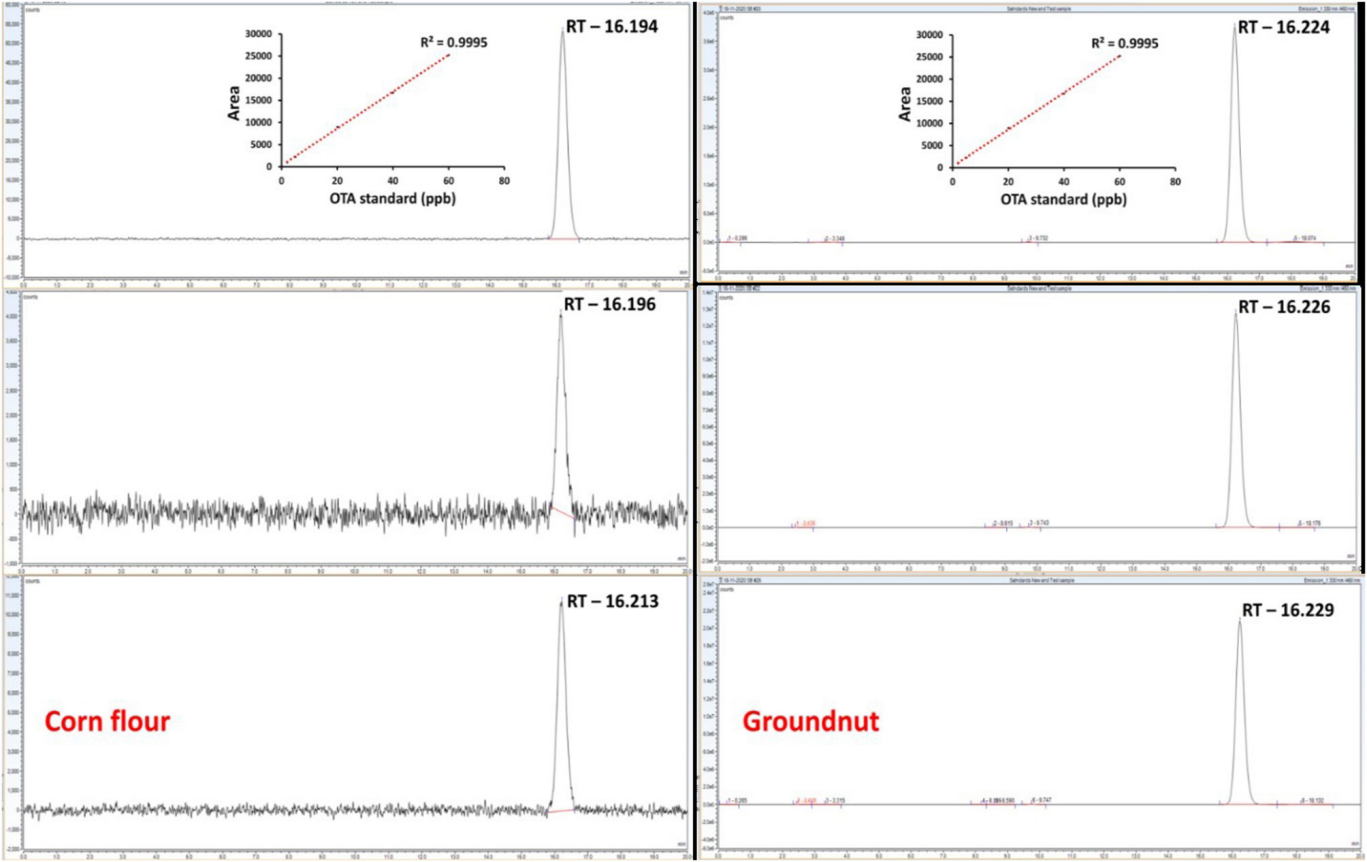
Comparison of the proposed aptamer and salt-induced AuNPs-based colorimetric OTA sensor with conventional HPLC-based detection in spiked corn and groundnut samples.

However, the HPLC approach requires several steps, such as sample extraction (2–4 h) and a single sample run of 20 min for every single sample (Figure 6). Importantly, the real sample data demonstrated that even though HPLC detection was able to detect OTA with a better detection range from 2 to 60 μg/mL of concentrations, it required multiple extraction and cleanup procedures and a sample run of a minimum 20 min along with the requirement of trained personal which were not required for the aptamer and salt-induced AuNP-based colorimetric OTA sensor developed in this study, thus increasing the feasibility of the developed colorimetric sensor than HPLC detection assays for its industrial use in on-site detection applications.

## Conclusions

In this study, a paper-based μPADs was developed for the detection of OTA in corn and groundnut. We also showed a method for quantitative detection of OTA in real food samples at low concentrations up to 95.69 ng/mL. The detection technique was based on the target-induced aggregation of AuNPs, the most common method for colorimetric detection. The colorimetric detection of the toxin was possible upon incubation of 5 min as the detection time of the developed assay. The linear equation observed for the absorption peak ratio (A_630/520_) was illustrated as a function of OTA concentration in spiked food samples in the case of groundnut and corn flour. The developed μPADs has a potent ability to be used for the rapid detection of OTA in many other food samples, including corn and groundnut. The proposed μPADs was not only simple but also cost-effective, rapid, and specific devoid of using any antibody or sophisticated enzymes.

## Data Availability Statement

The original contributions presented in the study are included in the article/supplementary material, further inquiries can be directed to the corresponding authors.

## Author Contributions

SG: conceptualization. SG and SS: methodology, supervision, project administration, funding acquisition, and resources. DS and VKB: software and writing-original draft preparation. DS, SS, and AAK: validation. DS: formal analysis and visualization. SG, AAK, AA, and SS: investigation. DS and AAK: data curation. SG, AA, and SS: writing-review and editing. All authors have read and agreed to the published version of the manuscript.

## Funding

SG, SS, and DS would like to thank NIAB core grant (C0038) and DBT-Ramalingaswamy Research grant (BT/RLF/Re-entry/20/2017, awarded to SS) for support to carry out the research work. SG, DS, VKB, and SS designed and performed the experiments. AAK's work is funded by the Researcher supporting Project (No. RSP-2021/339), King Saud University, Riyadh, Saudi Arabia.

## Conflict of Interest

The authors declare that the research was conducted in the absence of any commercial or financial relationships that could be construed as a potential conflict of interest.

## Publisher's Note

All claims expressed in this article are solely those of the authors and do not necessarily represent those of their affiliated organizations, or those of the publisher, the editors and the reviewers. Any product that may be evaluated in this article, or claim that may be made by its manufacturer, is not guaranteed or endorsed by the publisher.
